# Role of vascularised fibula graft in the surgical management of radiation-induced midshaft femoral fractures. Case report and literature review

**DOI:** 10.1186/s12957-024-03616-x

**Published:** 2024-12-20

**Authors:** Monique Khasin, Genevieve M. Darcy, Eldon Mah, Claudia Di Bella

**Affiliations:** 1 Department of Orthopaedic Surgery, Sarcoma Unit, St Vincent’s Hospital Melbourne, 41 Victoria Parade, Fitzroy, VIC 3065 Australia; 2 Department of Plastic Surgery, St Vincent’s Hospital Melbourne, 41 Victoria Parade, Fitzroy, VIC 3065 Australia; 3 Department of Surgery, The University of Melbourne, 29 Regent St, Fitzroy, VIC 365 Australia

**Keywords:** Free vascularised fibular graft, Orthopaedics, Non-union, Sarcoma, Intramedullary nailing, Pathological fracture

## Abstract

**Background:**

Post-radiation fractures (PRF) are a recognised complication of radiation treatment for soft tissue sarcomas. They have a low incidence and typically occur up to 5 years following treatment, more commonly affecting the pelvis, ribs and femur. Due to radiation-induced changes in bone, PRFs typically require more complicated intervention compared to post-trauma fractures, however, limited literature exists, particularly in regards to mid-shaft femoral PRFs. We report a case of a mid-shaft femoral PRF managed with a modified onlay free vascularised fibular grafting (FVFG).

**Case presentation:**

A 40-year-old male with a history of left quadriceps clear cell sarcoma successfully treated with wide local excision, chemotherapy and radiotherapy 18 years prior presented with a displaced oblique pathological fracture of his left femoral shaft. He was initially treated operatively with intramedullary nailing, however, repeat imaging at the one-year post-operative review demonstrated persistent hypotrophic non-union of the fracture. 16 months following the initial fracture, the patient underwent further surgical intervention with implantation of a modified onlay FVFG to the anterior aspect of the distal femur without nail removal. One-year post-revision, the patient was pain-free with normal mobility and imaging of both the graft and fracture site demonstrated complete union.

**Conclusion:**

Despite their operative complexity, we suggest that FVFGs should be considered for treating non-union of mid-shaft femoral PRFs due to their ability to promote healing and bone union in irradiated bone. Here we describe an original technique of a modified onlay FVFG which can be used in PRFs, and we have put this technique in the context of the current literature in FVFG.

## Introduction

Radiotherapy is used commonly in the treatment of soft tissue sarcomas in both the pre- and post-operative setting. Despite its effectiveness in improving oncological outcomes, recognised complications from radiation therapy include delayed bone and soft tissue healing, infection, osteonecrosis and post-radiation fractures (PRF) [1]. Unlike most fractures, PRFs can occur with minimal or no trauma [2–5].

PRFs typically occur at the original site of radiotherapy treatment. They occur usually within a 5-year period of initial treatment, although there have been documented cases of PRFs occurring up to 25 years post therapy [2, 3, 5–7]. Anatomical sites which are at a higher risk of PRF are the ribs, pelvis and femur [8]. The documented incidence of PRFs varies between 1.2 and 22% [2, 9, 10] with a recent systematic review reporting an incidence of 3% [8]. There are a number of factors associated with PRF including age, gender, size and dose of radiation, circumferential radiation and chemotherapy [3, 4, 7, 9, 11]. Tumours located in the anterior compartment of the thigh and periosteal stripping at the time of surgery have an increased risk of subsequent PRF of the femur [4, 9].

At the cellular level, exposure to radiation causes bone deterioration via increased osteoclast and reduced osteoblast activity and adipocyte filtration within the bone marrow [12, 13]. This may be in part due to changes in the differentiation potentials of bone marrow mesenchymal stem cells [13]. Architecturally, radiation exposure can also result in reduced bone vascularity [14]. These radiation-induced changes lead to increased risk of non-union, fractures and infection [15].

As such, PRFs commonly require more complicated treatment intervention compared to post-trauma fractures [15]. In a study by Helmstedter et al., 45% of PRFs resulted in non-union following surgical intervention, some of which involved intramedullary nailing, while another study by Lin et al. resulted in non-union in 5 out of 9 PRFs following treatment [3, 16]. A necessity for multiple follow-up surgeries is not uncommon, further increasing risk of infection and amputation [4, 8, 15]. At this stage, limited literature exists describing interventional options for mid-shaft femur PRFs. IMN, open reduction-internal fixation (ORIF) and vascularised fibular grafting have all been described with variable success in the context of PRFs, however no standardised method of treatment has been defined.

We report a case of a mid-shaft femoral PRF managed with a modified onlay free vascularised fibula graft (FVFG) due to non-union following initial intramedullary fixation and offer a treatment management solution for future PRFs.

## Case report

A 40-year-old male was brought into the emergency department via ambulance with left leg pain after collapsing whilst jogging. The patient was a non-smoker had a past history of left quadriceps clear cell sarcoma 18 years prior which at the time was managed with wide local excision which included resection of approximately 85% of the quadriceps. Adjuvant chemotherapy with 5 cycles of Epirubicin and Ifosfamide and external beam radiotherapy with a total of 60 Gy in 33 fractions was performed postoperatively. Initial imaging demonstrated a displaced oblique pathological fracture of his left femoral shaft and the patient was managed initially with analgesia and placed into skin traction. The fracture was investigated further to exclude a new malignant process. Positron emission tomography (PET) scan was performed followed by a computerised tomography (CT)-guided biopsy which confirmed the diagnosis of radiation-induced pathological fracture without any evidence of local recurrence. A long recon femoral intramedullary nail (IMN) was inserted and locked to stabilise the fracture and allow the patient to weight-bear. Post operatively, the patient was allowed to partial weight bear up to 50% on the left leg for six weeks.

**Fig. 1:**
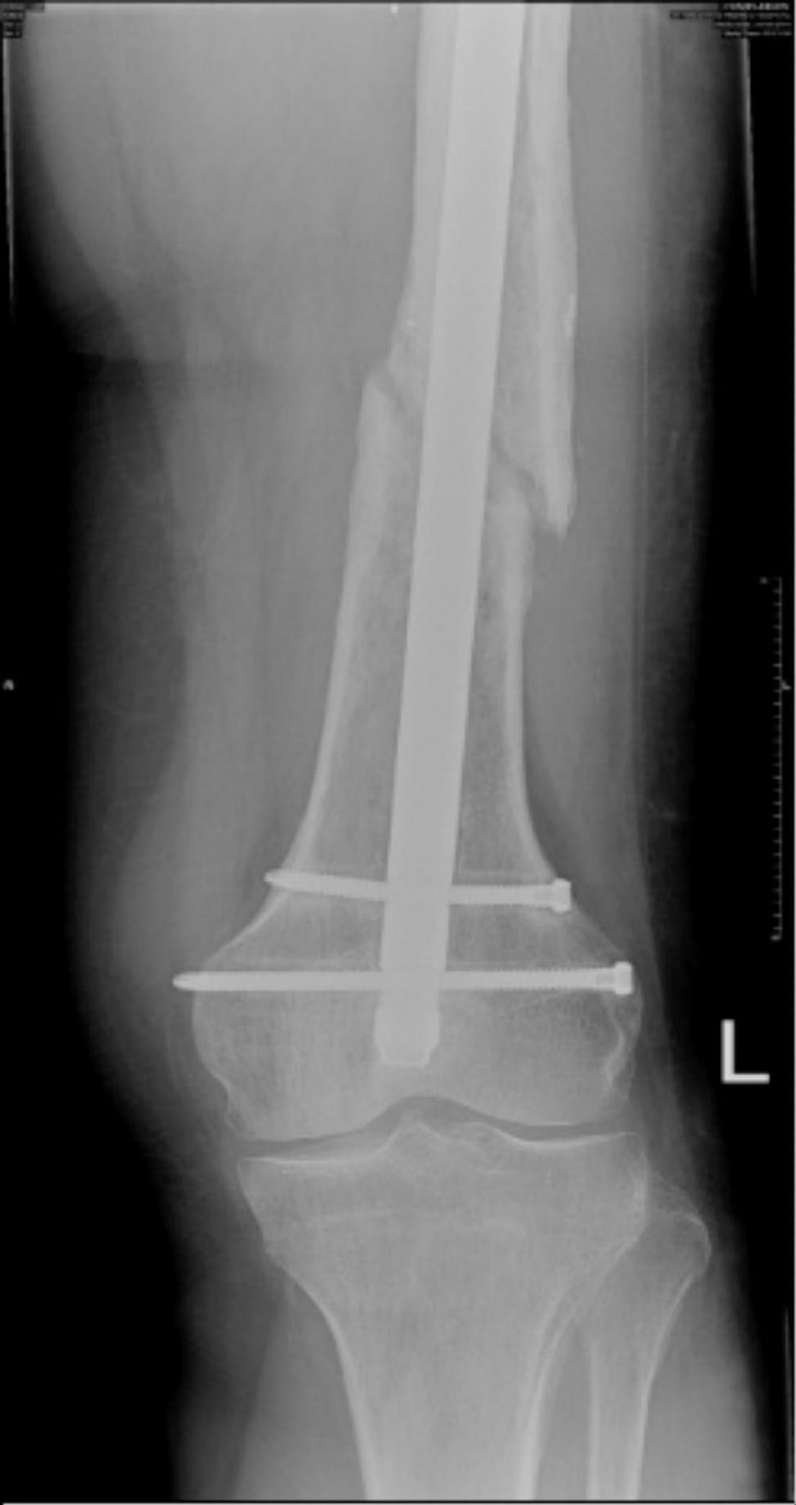
AP Radiograph demonstrating non-union of pathological left femoral shaft fracture and broken distal crossbolt. 2020

The patient was reviewed at six weeks and three months post-operatively and weightbearing status was progressed to weight-bearing as tolerated at the six-week review. At three months the patient was able to bear weight on his left leg with the aid of a single crutch, and was pain-free when walking, with knee range of movement returned to pre-surgery levels. Incomplete bone union was observed on X-rays performed at three months despite initial partial callous formation. Clinically at one year post-operatively the patient was able to walk without gait aids and had minimal pain. However repeat imaging demonstrated persistent hypotrophic non-union of the fracture (Fig. 1). Due to the concern about eventual failure of fixation the patient was referred to the plastic surgery team for consideration of a FVFG.

Work-up included a CT angiogram to assess vascularity around the fracture which demonstrated normal arterial vascular anatomy with no stenoses or significant calcification. On review at fourteen months post-operatively it was noted that one of the distal crossbolts had broken and the nail had begun to dynamise. At sixteen months post-initial fracture the patient underwent further surgical intervention.

**Fig. 2:**
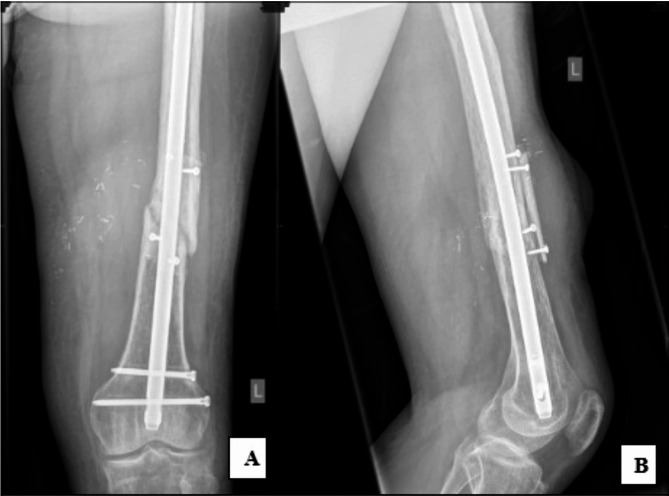
Post-operative AP (**A**) and Medial (**B**) radiographs demonstrating implanted onlay free vascularised fibular graft. The fibula is implanted around the antero-medial femur, with one half anterior and another half medial. 2020

**Fig. 3:**
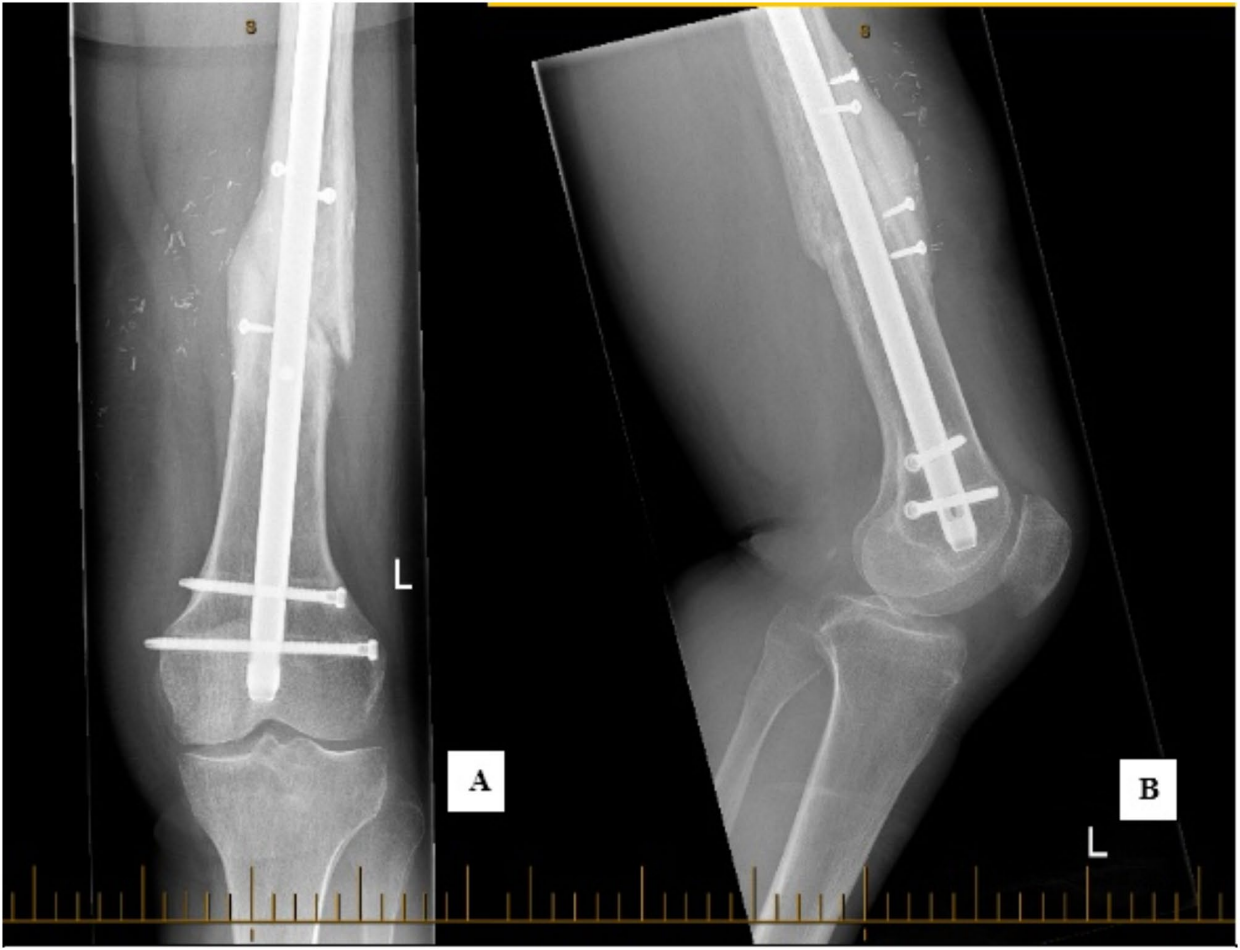
AP (**A**) and Lateral (**B**) Radiographs demonstrating union of femoral shaft fracture 3 years following onlay FVFG. 2023

The fracture site was debrided with the absence of bleeding bone noted initially and dead fibrous tissue was removed. The broken distal crossbolt was removed and a new crossbolt was inserted through the distal static hole in the IMN. Implantation of a modified onlay FVFG to the anterior aspect of the distal femur was performed using a 7 cm strut of distal bone from the right fibula and applied onlay without the removal of the nail. (Fig. 2A-B). The modified technique of the onlay involves splitting the fibula in half longitudinally as “open book” so that rather than simply placing the fibular against the femur (cortical bone to cortical bone), the “cut-surface” (i.e. the cortical and medullary bone) of the fibula are in direct contact with periosteum stripped irradiated femur fracture. The entire fibula was used. The flap was harvested with a skin pedicle and recipient vessels of the anastomoses were branches of the superficial femoral artery. The graft was secured with four 3.5 mm cortical screws. Following implantation, the fibula is covered with periosteum. No bone graft or substitute was utilised. The skin flap was used to monitor the survival of the bone graft. Post-operative instructions included protected weight-bearing of 50% with the support of crutches and use of a CAM Boot for four weeks on the right leg. At six weeks post-revision, the patient had complete wound healing and remained pain-free. X-ray demonstrated interval callous formation and the patient was continued on 50% weight-bearing restriction to allow for the biological processes to continue. Three-years post-revision, both the graft and fracture site demonstrated complete union on imaging (Fig. 3). The patient was pain-free with normal leg mobility and was able to perform low-impact exercise, including being able to walk and run un-aided.

## Discussion

Traditional fracture treatments, either operative or non-operative, rely on bone to have sufficient vascularity and cellularity for the healing process to occur, something which irradiated bone lacks [17]. Considering the cellular and architectural changes that occur, rates of union for PRFs are significantly lower, with additional risk of infection and amputation [4, 8, 15]. Currently no consensus for the gold standard treatment exists, partially due to the relatively low number of PRFs. The prescribed intervention is generally dependent on the experience and preferences of the operating surgeon. There is also limited literature that specifically focuses on mid-shaft femoral fractures [18]. Intramedullary nailing, ORIF, arthroplasty and vascularised fibula grafting (VFG) have all been described with variable success in the context of PRFs.

Both femoral intramedullary nailing and ORIF are a mainstay of treatment for mid-shaft femoral fractures [5]. Whilst these are common and appropriate treatments for healthy bone, the microvascular changes in irradiated bone lead to higher rates of non-union, ranging around 45-82% in multiple studies, as well as infection and metalware failure [3, 16, 19]. In comparison, some studies investigating closed, non-pathological femoral fractures have demonstrated non-union rates ranging from 2.8 to 14% [20–22]. While the union of non-pathological femoral fractures can be predicted within 4 months post-operatively [23], time to union in PRFs are often delayed and vary widely, with several studies identifying success within 9–12 months [24–26] In cases of persistent non-union further operative intervention is indicated. Revision ORIF with the implantation of supplemental bone grafting in cases where there is a bony defect is a commonly utilised procedure [5]. Bone grafting is utilised as an adjunct to facilitate osseous union via osteoinductive, osteoconductive and/or osteogenic mechanisms [21]. There are a variety of methods used which aim to stimulate bone healing including bone grafts, bone substitutes and orthobiologics. Bone grafting includes the use of autologous bone and substances such as platelet rich plasma (PRP), cortical and cancellous bone allograft as well as demineralised bone matrix (DBM). Autologous bone graft has been considered a gold standard due to its tissue compatibility and bone healing potential, however is associated with disadvantages such as donor site complications [27]. Allografting removes the potential for donor site complications however in most instances does not contain osteogenic properties [28]. Synthetic bone substitutes are an alternative option which are low risk and widely availability however has a limited role in fracture healing due to their primarily osteoconductive nature [27]. Recently, osteogenic growth factors such as bone morphogenetic proteins (BMP) have been utilised to augment fracture healing via osteoconductive and osteoinductive means, however more work is being done to understand their efficacy [27]. Each method has distinct advantages however only vascularised fibula grafting allows preservation of osteoprogenitor cells and subsequent osteogenic potential [27].

In cases where there is poor vascularity at the recipient site, such as in PRFs, previously discussed methods of bone grafting do not guarantee union [16]. A possible surgical technique for the treatment of non-union in radiation-induced pathological fracture is the implantation of an autologous FVFG. FVFGs involve implantation of an autologous live fibula to the fracture, a complex and prolonged procedure which requires a multi-disciplinary team with a specialised skillset. Specifically, these can include inlay and onlay strut grafts. Onlay grafts typically involve laying the donor bone along the host bone to promote callus formation while inlay grafts are constructed by creating a defect within the cortex of a bone with the graft then inserted into the defect. Onlay grafts can also be utilised as single or dual cortical grafts and are often a useful bone inset option for diaphyseal fractures with persistent non-union [29].

FVFGs have been utilised as the treatment for non-union or in cases where autologous bone grafting fails [5]. Unlike non-vascularised fibular grafts, FVFGs enable a remodelling process with notable hypertrophy to the recipient-graft site [30], and promote elevated osteocyte survival and maintenance of microcirculation [31]. Importantly, it has been demonstrated that, using vascularised bone grafts, union can be achieved in an irradiated bone host [17]. The fibula is favourably used as a bone graft due its size, straight shape and vascular supply, suiting pathologies of both the upper and lower extremity [32, 33] and has been noted to provide grafting potential for defects up to 26 cm long [34]. Survival of the bone can be on endosteal blood supply (i.e. fibular nutrient artery) and on periosteal blood supply [30]. As a general rule, attempt to save both vascular supplies is recommended, however often periosteal supply can be sufficient. FVFGs can be harnessed for a variety of pathologies including defects caused by tumour, infection, trauma, avascular necrosis and congenital defects [30, 33].

FVFGs have demonstrated to be effective to varying extents in the treatment of non-union in post-radiation pathological fractures. Within the current literature there are several studies that have looked at the outcomes of FVFGs in multiple contexts, including PRFs. However, of the available literature, techniques used vary widely, adding to the uncertainty about what the ideal treatment method should be. In our case, the pre-existing IMN was left in situ, and the split FVFG has been implanted as an onlay graft with single cortical screws, allowing for direct contact between two cortical bones (fibula endosteal bone to femur cortical bone) with fibular periosteum wrap at the fracture site to promote healing. Splitting the fibula provides an increased cortex-to-cortex contact between fibula endosteal bone and femur cortical bone, therefore increasing the biology at the fracture site. No additional bone graft or bone stimulating factors or adjuncts were used.

We performed a literature review looking at the varying techniques of free vascularised fibula grafting which have been described in the setting of radiation induced pathological fractures.

Duffy et al. [24] utilised onlay FVFG in 18 fractures combined with additional cancellous autogenous bone grafting from the iliac crest at the proximal and distal junctions of the graft in 18 radiation-induced fractures, reporting union in 16 within an average time of 9.4 months, with fourteen of these fractures being located in the femur. Of these fourteen patients, the initial fixation method was maintained in seven patients, and the metalware was exchanged in the remaining 50%. Ultimately, thirteen patients were disease and pain-free, one had some degree of functional impairment not impacting on lifestyle, two required some level of walking support with function impacted by ongoing pain, one had a failure of treatment, and one patient required an above knee amputation for recalcitrant non-union. Muramatsu et al. [35] used VFGs to treat recalcitrant non-union and large bone defects following tumour resection. Single inlay FVFG was transferred in seven patients, double FVFG transfer in seven patients and twin-barreled (folded) FVFG transfer was performed in three patients based on a single vascular pedicle. In cases with double fibula grafting, one was placed as inlay (intramedullary) graft and another was used as onlay graft. 23 out of 24 FVFG were successfully transferred, and 15 of the 16 reconstructed femurs achieved successful bone union within a two-year period.

Houdek et al. [25] retrospectively compared the rates of union of 109 free fibular grafts fixed with locking and traditional techniques for radiation-induced non-union. The fibular graft was fixed either using a locking plate spanning the entire graft and graft-recipient site junctions (in 27 patients), or by one of a number of other techniques including compression lag screws at the proximal and distal docking site (in 66 patients), non-locking compression plating either spanning the entire graft (in seven patients) or only at the proximal and distal docking sites (in six patients), external fixation (in two patients), or an IMN (in one patient). Union was ultimately achieved in 70% of patients within a 10-month mean. 91% of patients went on to achieve overall union. In 63 patients (58%) the fibula was used as an onlay graft; in the remainder it was telescoped between the proximal and distal ends of the recipient site. No surgical factor, including the use of locked fixation or supplementary cortico-cancellous bone grafts increased the rate of union. A history of smoking was found to be significantly associated with a risk of non-union.

Friedrich et al. [26] looked at 25 patients who underwent an onlay vascularised fibula flap for long bone pathological fracture non-union, 21 of which were post-radiation therapy, affecting the humerus, femur and tibia. 21 patients achieved bone union, within an average time of 11 months. Tibbo et al. [31] reviewed the outcomes of 23 patients who underwent free vascularised fibular flaps for the treatment of radiation-associated femoral non-unions. All patient had undergone at least one previous surgical procedure to treat their femoral fracture that had resulted in non-union. The fibula was fixed as an onlay graft using lag screws in all cases; additional fixation was obtained with an IMN (*n* = 19), blade plate (*n* = 2), dynamic compression plate (*n* = 1), or lateral locking plate (*n* = 1). Post FVFGs the union rate was 78% at a mean of 13 ± 6 months with a complication rate of 13%. In addition to FVFG, all 23 patients underwent simultaneous autogenous bone grafting. There was no difference in the union failure rates between fixation methods.

To our knowledge, this is the first report of a modified onlay FVFG where the fibula is longitudinally split in half to allow for cancellous to cortical bone contact.

## Conclusion

PRFs of bone are an uncommon complication of radiotherapy treatment. PRFs located in the mid-shaft of the femur are even less common, and scarcely described in the literature. As such, there is a lack of consensus as to what the most appropriate operative technique is. The goal of treatment is to stabilize the fracture to allow weight-bearing whilst mitigating the risk of complications such as non-union and post-operative infections. FVFGs are a viable operative option for the treatment of non-union of mid-shaft femur PRF due to their ability to promote healing and bone union in the context of an irradiated bony environment, and should be considered as an option in the initial treatment of PRFs. The operative complexity and individual patient factors, including vascular health, remain a consideration for FVFG procedures. It can be suggested that there is a role for utilisation of FVFG as first line treatment for patients who are high risk of non-union with conventional methods of fracture fixation. Whilst traditional inlay and onlay FGFV techniques have been described with various degrees of success, to our knowledge this is the first description of this original technique, where the split fibula with cancellous to cortical bone contact wrapped in periosteum has the theoretical advantage of faster and higher union rate. This case report demonstrates the successful utilisation of a modified onlay FVFG for treatment of non-union post treatment of a femoral shaft PRF. We believe this is an important addition to the literature and that further studies with increased patient numbers will be required to evaluate its role compared to the more traditional approaches.

## Data Availability

No datasets were generated or analysed during the current study.

## Abbreviations

BMP: Bone morphogenetic proteins
CT: Computerised tomography
DBM: Demineralised bone matrix
FVFG: Free vascularised fibular graft
IMN: Intramedullary nail
ORIF: Open reduction-internal fixation
PET: Positron emission tomography
PRF: Post-radiation fracture
PRP: Platelet rich plasma
VFG: Vascularised fibular graft
